# Time to reimbursement of novel anticancer drugs in Europe: a case study of seven European countries

**DOI:** 10.1016/j.esmoop.2023.101208

**Published:** 2023-04-06

**Authors:** H.C. Post, T. Schutte, M.G.H. van Oijen, H.W.M. van Laarhoven, C.E.M. Hollak

**Affiliations:** 1 Department of Oncology, Amsterdam UMC Location University of Amsterdam, Amsterdam, The Netherlands; 2 Cancer Center Amsterdam, Cancer Treatment and Quality of Life, Amsterdam, The Netherlands; 3 Platform ‘Medicijn voor de Maatschappij’ (Medicine for Society), Amsterdam, The Netherlands; 4 Department of Oncology, Amsterdam UMC Location Vrije Universiteit Amsterdam, Amsterdam, The Netherlands; 5 Department of Endocrinology and Metabolism, Amsterdam UMC Location University of Amsterdam, Amsterdam, The Netherlands

**Keywords:** anticancer medicines, drug access, inequality, reimbursement, regulatory approval

## Abstract

**Background:**

Time to reimbursement (TTR) of new anticancer medicines differs between countries and contributes to unequal access. We aimed to investigate TTR of new anticancer medicines and explore factors influencing the reimbursement process in seven high-income European countries.

**Materials and methods:**

We carried out a retrospective case study of anticancer medicines with European Union Market Access (EU-MA) and a positive Committee for Medicinal Products for Human Use opinion from 2016 until 2021 with subsequent national reimbursement approval (NRA). The National Health Technology Assessment (HTA) and reimbursement websites of Germany, France, UK, the Netherlands, Belgium, Norway and Switzerland were used to identify TTR, defined as time from EU-MA to NRA. Additionally, we investigated medication-, country-, indication- and pharma-related factors potentially influencing TTR.

**Results:**

Thirty-five medicines were identified for which TTR ranged from -81 days to 2320 days (median 407 days). At data cut-off, 16 (46%) were reimbursed in all seven countries. Overall, the shortest TTR was in Germany (median 3 days, all medicines reimbursed <5 days). The time limit for reimbursement of 180 days stated by the Council of European Communities after the EU-MA (EU Transparency Directive) was met for 100% of included medicines in Germany, 51% in France, 29% in the UK and the Netherlands, 14% in Switzerland, 6% in Norway and 3% in Belgium. The TTR was significantly different between countries (*P* < 0.001). In multivariate analysis, factors associated with shorter TTR were higher gross domestic product (GDP), absence of a pre-assessment procedure and submission by a big pharmaceutical company.

**Conclusions:**

TTR of anticancer medicines varies significantly between seven high-income European countries and leads to inequality in access. Among explored medication-, country-, indication- and pharma-related factors we found that a high GDP, the absence of a pre-assessment procedure and submission by big pharmaceutical companies were associated with shorter TTR.

## Highlights

• Time from approval to reimbursement for new anticancer medicines is long, even in most high-income European countries.
• Large differences exist for TTR between seven high-income European countries.
• Factors contributing to a short TTR are no pre-assessment procedure, high GDP and Big-12 pharmaceutical company medicines.

## Introduction

Novel anticancer medicines are introduced every year for treatment of patients with cancer. DeIays and inequalities in access to anticancer medicines are seen worldwide, and potentially lead to a substantial loss of quality of life and life years.^1^ For this reason, it is important for patients and their prescribers that inequality in access is as little as possible. Patients’ access to medication in the European Union (EU) and some countries of the European Economic Area (Iceland, Liechtenstein and Norway) starts with centralized authorization decisions on quality, safety and efficacy by the Committee for Medicinal Products for Human Use (CHMP) of the European Medicines Agency (EMA). After the EMA advices on marketing authorization (MA), the European Commission makes the formal decision to grant MA. Once approved and EU-authorized, reimbursement must be agreed at a national level before therapies are accessible for all (cancer) patients in an EU member state. Although the Council of European Communities established by the EU Transparency Directive^2^ has set 180 days as the deadline for member states to make pricing and reimbursement decisions for innovative medicines, previous research has shown that the period between EU-MA and a positive reimbursement decision differs between countries.^3^

Several factors have been suggested to affect time to reimbursement (TTR), and they can be divided into medication-, country-, indication- and pharmaceutical company-related factors. Medication-related factors described are: the level of evidence for clinical benefit,^4^, ^5^, ^6^ the level of innovation/therapeutic importance of anticancer medicines^7^ and the product price.^8^ Country-based factors that may affect TTR are the presence or absence of an assessment procedure before reimbursement and the gross domestic product (GDP).^7^, ^8^, ^9^, ^10^ Indication-related factors that might influence TTR are orphan status and rarity of the disease and a high unmet medical need for treatment.^11^ A pharmaceutical company-related factor that might influence the TTR is the size of the company.^7^ Of note, these factors have not yet been quantitatively investigated or are still ambiguous.

The aim of this study was to assess the TTR to novel anticancer medicines for solid tumors in seven high-income European countries and its association with medication-, country-, indication- and pharmaceutical company-related factors potentially influencing the reimbursement process.

## Materials and methods

### Study design

We carried out a retrospective case study to investigate the TTR after EU-MA of new anticancer medicines for solid tumors in high-income European countries. The TTR was defined as the time (in days) between EU-MA and registered/announced date of reimbursement. A difference in TTR of >30 days was considered as clinically relevant. In addition, we investigated medication-, country-, indication- and pharmaceutical company-related factors which might influence TTR.

### Selection of medicines

Firstly, we identified the approved new anticancer medicines from the meeting highlights of the EMA CHMP, from January 2016 to January 2021.^12^ Products under the headings ‘positive recommendations on new medicines’ and ‘medicines recommended for approval’ and ‘recommend granting marketing authorizations for cancer medicines’ were eligible for inclusion. We also identified anticancer medicines that received conditional authorization status by EMA with requirement for subsequent data to be provided before full standard authorization can be granted. Supportive products developed for treatment of symptoms caused by cancer or cancer treatment, for example, pain, nausea, vomiting and chemotherapy-induced neutropenia, were excluded. Cancer medicines for hematological malignancies and generic or biosimilar medicines were also excluded. Cancer medicines that were already on the market and now available in a different formulation (e.g. subcutaneously instead of intravenously) were excluded as were medicines withdrawn after initial or accelerated approval.

### Selection of countries

A convenience sample of seven high-income European countries were examined. For these countries, except for the Netherlands, the data on TTR were publicly available. In all of these countries, the state plays a major role in the pricing and reimbursement process of medicines. At the time of our study, the UK was an EU member. The time of MA for all these countries was the same, except for Switzerland where a national approval procedure is in place by Swissmed.

In brief, these European countries, except for Germany, follow a comparable national reimbursement approval (NRA) procedure: firstly, a pharmaceutical company should submit documentation on drugs’ safety and effectiveness, their relative value, impact on services and organization of health care, cost-effectiveness analysis and budget impact estimates. Secondly, the application is reviewed by an independent expert committee. Thirdly, when approved, price negotiations can start and only after agreement, reimbursement has been arranged. In Germany, price and reimbursement regulation does not play a role in initial launch of therapies, but products are scrutinized after 1 year.^13^

### Data collection

The relevant Health Technology Assessment (HTA) and reimbursement websites for each country were used to identify decision information and dates: date of inclusion in the relevant positive list, the date of the publication of an HTA recommendation or a pricing and reimbursement (P&R) commissioning decision, depending on the country were used (see Supplementary Appendix 1, available at https://doi.org/10.1016/j.esmoop.2023.101208, for details of websites visited and the retrieval process of reimbursement dates). Data were extracted by one researcher and 20% of the TTR data were checked by a second researcher. Eventual discrepancies were discussed together to reach agreement.

### Medication-, country-, indication- and pharmaceutical company-related factors

As a potential medication-related factor of influence, we used the level of evidence as scored in the European Society Medical Oncology-Magnitude of Clinical Benefit Scale version 1.1 (ESMO-MCBS v1.1).^14^ The ESMO-MCBS v1.1. aims to determine the relative benefit of anticancer medicines based on their overall survival (OS), progression-free survival (PFS), response rate, toxicity and quality-of-life outcomes, such that they can be compared and their clinically meaningful benefit can be quantified.^15^ It is intended to facilitate EMA approval and serve as evidence for national and international clinical guidelines. We extracted the ESMO-MCBS in medicine-specific reports on the ESMO website and always adopted the latest published score on 27 December 2021. High benefit was defined as a score of A-B (in adjuvant and neo-adjuvant therapy setting) or >3 (in palliative setting). Low benefit was defined as any other score.^15^ Furthermore, the level of innovation/therapeutic importance of an anticancer drug was assessed.^7^ EMA can use an accelerated approval (AA) procedure for medicines that are considered to be of major interest for public health or therapeutic innovation. We compared TTR for EMA-AA-graded anticancer medicines to that of anticancer medicines with a regular assessment procedure.

Country-related factors were the presence or absence of a pre-assessment procedure and the GDP (as reported by EuroStat).^16^

Indication-related factors we examined were: the orphan status (yes/no) as reported by EMA, for non-orphan status the presence of an unmet medical need. For anticancer medicines with an unmet medical need (including orphan status), EMA has a conditional marketing authorization (CMA yes/no) procedure. These data were extracted from the medicine-specific European public assessment reports (EPAR) of EMA.^17^ Rare cancers were defined as cancers with an incidence of fewer than 5 in 100 000 persons per year.^18^ Size of the company was dichotomized according to the Big-12 pharmaceutical companies of the world.^19^ The database lock was 1 January 2023. Anticancer medicines that were not nationally reimbursed by that date were censored. Reported data are the censored data, unless explicitly indicated.

### Analysis

To evaluate TTR, we calculated the time interval in calendar days between the EU-MA date and the regulatory submission in each country for each drug. A difference in TTR of >30 days was considered clinically relevant. Descriptive statistics were used to describe median, range and interquartile range (IQR) of the TTR in the different countries and for the given subgroups, such as: ESMO-MCBS score (high/low), EMA-AA status (yes/no), GDP (euro), orphan status (yes/no), CMA (yes/no), company size (Big 12/non-Big 12) and pre-assessment procedure (absent/present). Given the anticipated non-normal distribution of TTR, non-parametrical tests were used, first to compare TTR distribution between all countries and then for pairwise comparisons between the different countries (Friedman’s two-way analysis of variance by ranks).

Survival analysis was used to analyze the TTR given that some medicines were not reimbursed at the date of database lock. In most other methods of analysis, this missing data would be excluded; however, the non-reimbursement up to a specific date is an outcome on its own. If a reimbursement decision was not available at the date of database lock, the observation was censored. Kaplan–Meier and Cox proportional hazard regression analysis were used to report hazard ratios (HR) and 95% confidence intervals (95% CIs) using univariate analysis to report differences between the absence and presence of a specific factor. Moreover, a multivariate Cox proportional hazard regression analysis reporting HR and 95% CI was used to describe the association between TTR and the factors together. For the analysis of factors within countries, Mann–Whitney *U* tests were used. For all statistical tests, a *P* value <0.05 was considered statistically significant. Given the multiple tests, a Bonferroni-corrected *P* value was used for the multiple paired comparisons (see Supplementary Appendix 2, available at https://doi.org/10.1016/j.esmoop.2023.101208). Statistical analyses were carried out using SPSS (IBM, version 28, Chicago, IL), and figures were created using GraphPad Prism v9.1.0 (GraphPad Software, San Diego, CA).

## Results

From January 2016 to January 2021, 36 new anticancer medicines received an EU-MA. One drug, olaratumab (Lartruvo), was withdrawn from EU-MA and therefore excluded from the analysis. A detailed flow diagram of the selection of included medicines is provided in Supplementary Appendix 1, available at https://doi.org/10.1016/j.esmoop.2023.101208. Thirty-five new anticancer medicines were evaluated. At data cut-off, 16 (46%) of the anticancer medicines were reimbursed in all seven countries. The median number of cancer medicines reimbursed in the examined countries was 28 (80%) with a range of 21 in Belgium (60%) to 35 in Germany (100%). One drug, paclitaxel micellar (Apealea), was only reimbursed in Germany. For the 16 medicines available in all countries, the pooled median TTR was 234 days (uncensored data, only available medicines). The reimbursement time limit of 180 days set by the EU Transparency Directive was met 100% (*n* = 35) in Germany, 51% (*n* = 16) in France, 29% (*n* = 10) in the UK and the Netherlands, 14% (*n* = 5) in Switzerland, 6% (*n* = 2) in Norway and 3% (*n* = 1) in Belgium.

**Figure 1:**
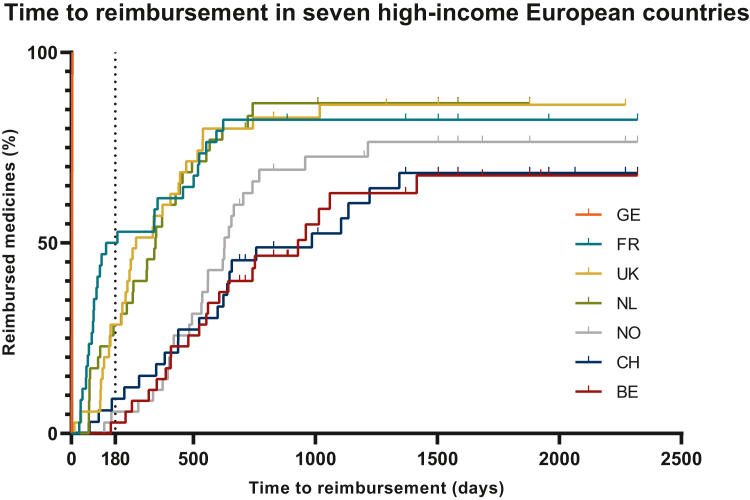
**Kaplan–Meyer curve of time to reimbursement in seven high-income European countries in days.** mTTR GE = 3 days, FR = 142 days UK = 265 days, NL = 345 days, NO = 628 days, CH = 689 days, BE = 742 days (mTTR and IQR specifically for each country see Table 1, Supplementary Appendix 3, available at https://doi.org/10.1016/j.esmoop.2023.101208). The dotted vertical line at 180 days indicates the Council of European Communities stated time limit for reimbursement of 180 days after EU-MA. The EU Transparency Directive reimbursement time limit of 180 days was met 100% (*n* = 35) in Germany, 51% (*n* = 16) in France, 29% (*n* = 10) in the UK and the Netherlands, 14% (*n* = 5) in Switzerland, 6% (*n* = 2) in Norway and 3% (*n* = 1) in Belgium. GE, Germany; FR, France; UK, the United Kingdom; NL, the Netherlands; NO, Norway; CH, Switzerland; BE, Belgium; IQR, interquartile range; mTTR, median time to reimbursement.

**Figure 2:**
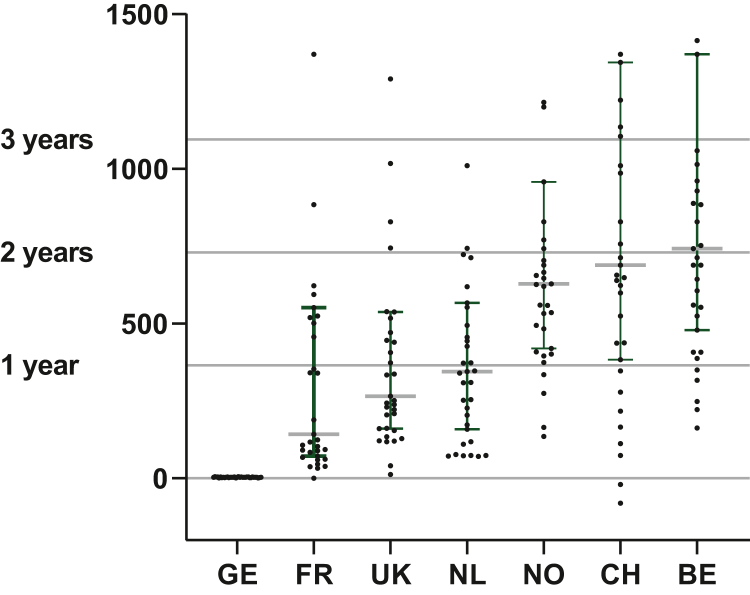
**Scatter dot plot of time to national reimbursement from EU-MA per reimbursed new anticancer medicine in the different countries.** Median (bar) and interquartile range (error bars, red) displayed. Statistically significant differences exist (tested with Mann–Whitney *U* tests) and after Bonferroni correction for multiple testing are indicated with an asterisk and described hereafter. GE-UK *P* < 0.001∗; GE-FR *P* < 0.001∗; GE-NL *P* < 0.001∗; GE-NO *P* < 0.001∗; GE-CH *P* < 0.001∗; GE-BE *P* < 0.001∗; FR-NO *P* < 0.001∗; FR-CH *P* < 0.001∗; FR-BE *P* < 0.001∗; UK-NO *P* = 0.006; UK-CH *P* = 0.009; BE *P* < 0.001∗; NL-NO *P* = 0.004; NL-CH *P* = 0.006; NL-BE *P* < 0.001∗. All statistical test results are described in Supplementary Appendix 2, available at https://doi.org/10.1016/j.esmoop.2023.101208. GE, Germany; FR, France; UK, United Kingdom; NL, the Netherlands; NO, Norway; CH, Switzerland; BE, Belgium.

The TTR in seven high-income European countries for each new anticancer medicine examined is shown in Figures 1 and 2, Table 1, Supplementary Appendices 3 and 4, available at https://doi.org/10.1016/j.esmoop.2023.101208. In all countries, except for Germany, there is a large range in TTR. In Germany, the median TTR was 3 days (0-5 days). The pooled median TTR for all studied drugs in all seven countries was 407 days. After Germany, France has the shortest median TTR of 142 days (IQR 72-552 days), followed by the UK with 265 days (IQR 160-538 days), the Netherlands with 345 days (IQR 158-567 days), Norway with 628 days (IQR 420-958 days), Switzerland with 689 days (IQR 383-1344 days) and Belgium with 742 days (IQR 479-1370 days). In Switzerland, for two anticancer medicines (6%) TTR was ahead of EU-MA. There was a statistically significant difference in median TTR between countries (*P* < 0.001, Friedman’s two-way analysis of variance by ranks). When studied in detail, a statistically significant difference persisted even after Bonferroni correction between Germany and all other countries, France versus Switzerland, Belgium and Norway and between UK and the Netherlands versus Belgium (Figure 2, Supplementary Appendix 2, available at https://doi.org/10.1016/j.esmoop.2023.101208).

**Table 1:** Time to national reimbursement from date of EMA marketing authorization per reimbursed new anticancer medicine in the different countries and specified sub-categories

Time to reimbursement in days							
		Number of censored/total *n*	Median	Min	Max	IQR 25th percentile	IQR 75th percentile
Country (GDP, 2019)	Germany (€3 473 350.00)	0/35	3	1	5	2	4
	France (€2 437 635.00)	6/35	142	0	2320	72	552
	United Kingdom (€2 526 615.20)	5/35	265	12	2270	160	538
	The Netherlands (€813 055.00)	5/35	345	71	1878	158	567
	Norway (€361 734.60)	9/35	628	135	2320	420	958
	Switzerland (€653 732.60)	12/35	689	−81	2320	383	1344
	Belgium (€478 238.90)	14/35	742	162	2320	479	1370
	Total	53/245	407	−81	2320	110	743
ESMO-MCSB	Low	133	427	1	2270	117	743
	High	91	340	−81	2320	91	594
Accelerated approval status	Non-accelerated approval	210	398	−81	2270	103	744
	Accelerated approval	35	494	2	2320	128	743
Orphan medicine status	Non-orphan medicine	182	379	−81	2320	93	657
	Orphan medicine	63	619	1	2270	124	1215
Conditional marketing authorization status	No conditional marketing authorization	168	398	−81	2320	121	944
	Conditional marketing authorization	77	444	1	1684	74	689
Company size: Big12	No	133	524	0	2320	128	1135
	Yes	112	270	−81	1370	81	623
Pre-assessment procedure	No pre-assessment procedure	35	3	1	5	2	4
	Pre-assessment procedure	210	509	−81	2320	222	884

Data are censored on 1 January 2023 for medicines not reimbursed up to this date. In the upper part of the table, the column ‘Censored/total *n*’ indicates the number of censored data points for the varying countries. The country-specific GDP is the 2019 GDP according to EuroStat^16^ which was extracted in August 2022. Data were complete for all included medicines (*n* = 35) in all countries (*n* = 7), thus 245 country–factor combinations, except for the ESMO-MCSB score (which included 224 combinations) due to a missing MCSB score for three medicines (and thus 21 combinations). Further details with factor distributions within the countries are provided in Supplementary Appendix 3, available at https://doi.org/10.1016/j.esmoop.2023.101208.

EMA, European Medicine Agency; ESMO-MCBS, European Society Medical Oncology-Magnitude of Clinical Benefit Scale; GDP, gross domestic product; IQR, interquartile range.

### Medication-related factors

**Figure 3:**
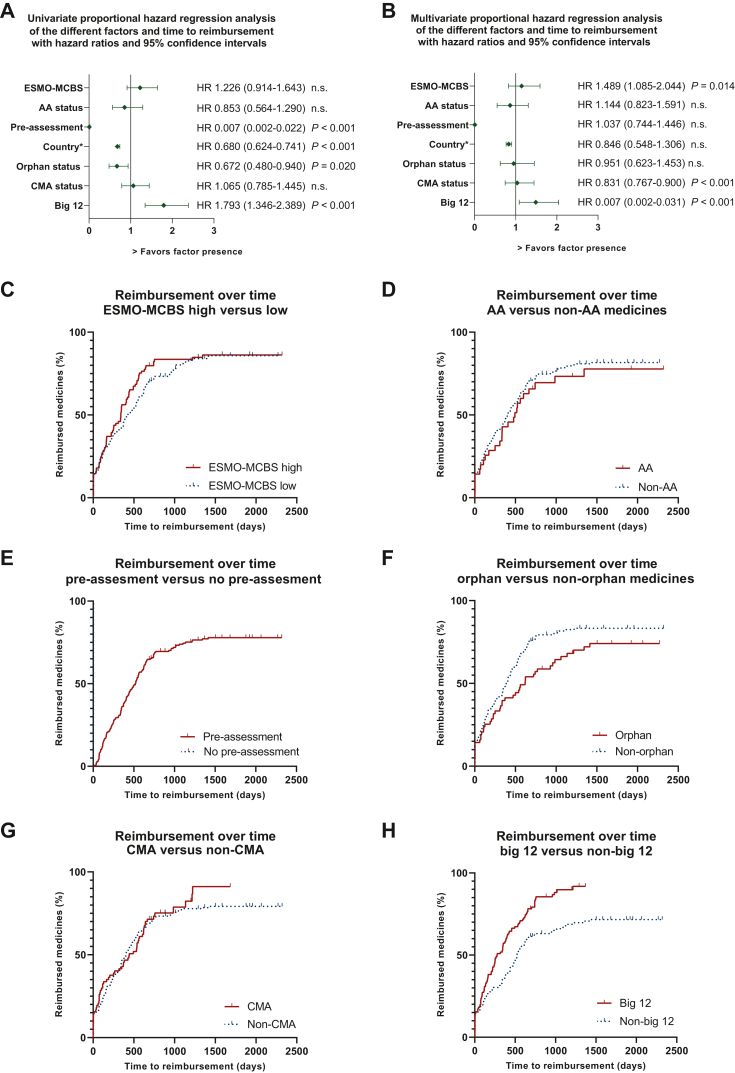
**Univariate and multivariate analysis of the different factors.** HR, 95% CI and IQR are displayed, together with Kaplan–Meyer curves for reimbursement over time and the different factors. In the Kaplan–Meyer curves, the two medicines registered in Switzerland before EMA-MA were set at 0 days to create the figure. For the factor Country (panels A and B), countries were sorted based on mTTR. In panel 3E, pre-assessment graft seems invisible because it overlaps the y-axis. Three drugs were not assessed by ESMO and no ESMO-MCBS score was available; these three medicines were omitted in the analysis of this factor.CI, confidence interval; ESMO-MCBS, European Society Medical Oncology-Magnitude of Clinical Benefit Scale; HR, hazard ratio; IQR, interquartile range; mTTR, median time to reimbursement.

Thirteen anticancer medicines (37%) had a high ESMO-MCBS score and nineteen anticancer medicines (54%) had a low ESMO-MCBS. Three drugs were not assessed by ESMO and no ESMO-MCBS score was available; these three medicines were omitted in the analysis of this factor. Overall, no statistically significant differences existed in TTR for ESMO-MCBS-high versus ESMO-MCBS-low scores [HR 1.226 (95% CI 0.914-1.643)] (log-rank, Figure 3). For anticancer medicines with a high ESMO-MCBS, the median TTR was shorter than that for cancer medicines with a low ESMO-MCBS in France, the UK, Norway, Switzerland and Belgium. Even though this was clinically relevant, in France (189 versus 107 days), in Norway (656 versus 536 days), in Switzerland (713 versus 438 days) and in Belgium (829 versus 643 days), it was not statistically significant.

**Figure 4:**
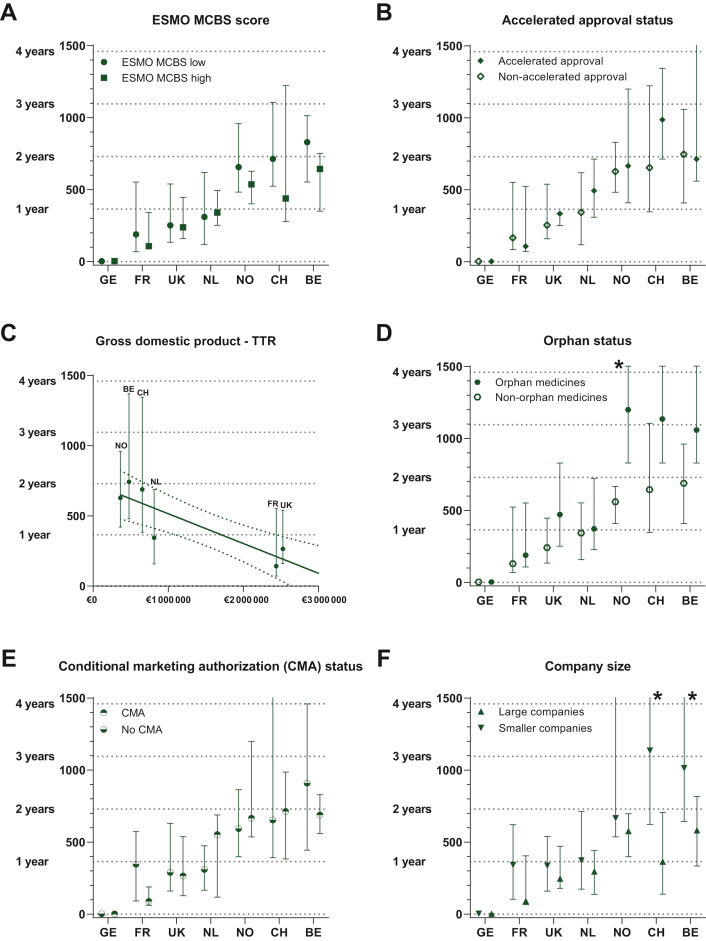
**Time to national reimbursement (TTR) from EU-MA per reimbursed new anticancer medicine in the different countries** (GE, Germany; FR, France; UK, United Kingdom; NL, The Netherlands; NO, Norway; CH, Switzerland; BE, Belgium) **within the specified sub-categories.** Median (bar) and IQR (error bars) are displayed for panels A, B, D, E, F. 95% confidence interval is displayed for panel C (dotted line). Significant differences after correction for multiple testing are displayed (∗) only in categories orphan status (for NO, *P* = 0.005∗) and company size (for CH, *P* < 0.001∗ and BE, *P* = 0.0044∗). Moreover, linear regression showed association between TTR and GDP and can be described in the linear function TTR = −0.0002119xGDP + 727.2 with a good fit (*R*^2^ = 0.8427; *P* = 0.0035). All statistical test results are described in Supplementary Appendix 2, available at https://doi.org/10.1016/j.esmoop.2023.101208. AA, accelerated approval; CMA, conditional marketing authorization; ESMO-MCBS, European Society Medical Oncology-Magnitude of Clinical Benefit Scale; GDP, gross domestic product; HR, hazard ratio; IQR, interquartile range.

Five anticancer medicines were rated by EMA through AA (Figure 4B). One was an orphan drug for a rare disease, submitted by a big pharmaceutical company. Four had a low ESMO-MCBS score and were submitted by smaller companies. Overall, no statistically significant differences exist in median TTR (mTTR) for AA status (absent versus present), with an HR of 0.853 (95% CI 0.564-1.290) (log-rank, Figure 3). Within countries, the mTTR in the presence or absence of AA status differed most in the Netherlands (non-AA 343 days versus AA 494 days) and Switzerland (non-AA 653 days versus AA 986 days); although probably clinically relevant, these differences were statistically insignificant.

### Country-related factors

In Germany, unlike the other countries in this study, health economic evaluations do not play a meaningful role in early benefit assessments and are not intended to be a regulatory threshold for market access based on the German Act on the Reform of the Market for Medicinal Products (AMNOG).^13^ Therefore, no drug is denied access to the market in Germany because of a poor cost–benefit ratio and in our study all the EU-approved anticancer medicines are reimbursed within 5 days. A statistically significant difference exists between Germany (the only country without a pre-assessment procedure) and other countries [HR 0.007 (95% CI 0.002-0.022, *P* < 0.001)] (univariate log-rank, see Figure 3).

A strong correlation was found between GDP and TTR (see Figure 4C, excluding Germany in this analysis, as Germany is an outlier being the largest country with the highest GDP and no pre-assessment procedure). Linear regression showed an association between TTR and GDP with a good fit (*R*^2^ = 0.8427; *P* = 0.0035). This can be explained such that for every €4720 increase in GDP, the median TTR shortens by 1 day. The median TTR of the Netherlands falls just outside the 95% CI and seems to have a slightly shorter median TTR than other countries.

### Indication-related factors

For indication-related factors, orphan status (Figure 4D) and CMA (Figure 4E) were analyzed.

A minority of 9 anticancer medicines (26%) were registered with an orphan drug designation, compared to 26 non-orphan medicines (Supplementary Appendix 3, available at https://doi.org/10.1016/j.esmoop.2023.101208). Overall, TTR for non-orphan medicines within the countries was shorter compared to orphan medicines [HR 0.672 (0.480-0.940, *P* = 0.020)] (Log-rank, Figure 3A). This difference was observed in all countries (Table 1); however, it was only statistically insignificant for Norway where TTR was 560 days versus 1200 days, (*P* = 0.005, paired Mann-Whitney *U* test).

Eleven anticancer medicines had a CMA (Figure 4E). Overall, no statistically significant differences exist in mTTR for CMA versus non-CMA medicines [HR 1.065 (0.785-1.445)] (Log-rank, Figure 3G). The mTTR of medicines with CMA compared to medicines without CMA within countries were all statistically insignificant, and shorter in four countries: Germany (2 days versus 3 days), France (89 days versus 347 days), UK (265 days versus 289 days) and Belgium (689 days versus 909 days). In the other countries, median TTR was longer in CMA versus non-CMA medicines: the Netherlands (444 days versus 310 days) and Norway (666 days versus 593 days) (Figures 4E and 3, Supplementary Appendix 3, available at https://doi.org/10.1016/j.esmoop.2023.101208).

### Pharmaceutical company-related factors

Almost half of the included medicines (*n* = 16, 46%) were submitted for approval by one of the Big-12 pharmaceutical companies. Overall, medicines submitted by the Big-12 pharmaceutical companies had an mTTR of 270 days versus 524 days for smaller companies [HR 1.793 (1.346-2.389)] (*P* < 0.001 log-rank) (Figure 3H, Table 1, Supplementary Appendix 3, available at https://doi.org/10.1016/j.esmoop.2023.101208). Within countries, statistically insignificant differences in TTR exist (Figure 4F, Supplementary Appendix 3, available at https://doi.org/10.1016/j.esmoop.2023.101208). The only statistically significant and notable differences were in Switzerland with an mTTR of 1135 days for smaller pharmaceutical companies and 365 days for the Big-12 pharmaceutical companies (*P* < 0.001) and in Belgium with an mTTR of 1014 days for smaller companies versus 583 days for the Big-12 companies (*P* = 0.004), both paired Whitney *U* tests. Notable differences in mTTR existed also in France (341 days versus 92 days), UK (337 days versus 247 days), the Netherlands (373 days versus 297 days) and Norway (666 days versus 577 days), albeit statistically insignificant.

### The combined factors

When all factors are combined using multivariate analysis, the factors that remained statistically significant are pharmaceutical company size [HR 1.489 (95% CI 1.085-2.044)] (*P* = 0.014, log-rank), the factor country [sorted low to highest median TTR, HR 0.831 (0.767-0.900), *P* < 0.001 log-rank] and the absence of a pre-assessment procedure [HR 0.007 (0.002-0.031), *P* < 0.001 log-rank] (Figure 3B).

## Discussion

The present study was designed to assess differences in TTR in high-income European countries and explore different medication-, country-, indication- and pharmaceutical company-related factors potentially influencing TTR. Based on the results of our study, TTR for new anticancer medicines is too long even in high-income European countries, except for Germany. TTR for new anticancer medicines differ considerably between countries and the major factors contributing to a shorter TTR are the absence of a pre-assessment procedure, a higher GDP and submission by Big-12 pharmaceutical companies. Hereafter, we will discuss the medication-, country-, indication- and pharmaceutical company-related factors.

Interestingly, no clinically relevant correlation between TTR and the level of clinical benefit (ESMO-MCBS v1.1) was observed as a medication-related factor. Results from previous studies on associations between TTR and the ESMO-MCBS are mixed: some observed a correlation with ESMO-MCBS,^6^ whereas others did not.^4^^,^^5^ The application of value frameworks, such as the ESMO-MCBS v1.1, could support the early identification of anticancer medicines with a perceived high clinical benefit and help to prioritize and support price negotiations and accelerate access.^20^^,^^21^ Next to the influence of national differences in using and accepting criteria of effectiveness,^10^ a higher level of clinical benefit might set a higher price level, resulting in difficult and extended price negotiations leading to a longer TTR. However, in a prior study, a higher level of clinical benefit was not significantly associated with treatment costs for anticancer medicines in four high-income European countries (England, France, Germany and Switzerland).^21^ The second medication-related factor was applications for AA status. AA status is meant for medicines of major interest for public health, particularly from the point of view of therapeutic innovation. AA by EMA reduces the timeframe to review an MA application by the CHMP from 210 to 150 days. Although the aim of EMA for anticancer medicines to grant AA status is accelerating access, in our study, in the multivariate analysis AA did not lead to a shorter TTR. In the univariate analysis, median TTR was shorter for Belgium (Δ34 days) and France (Δ59 days). In the UK, the Netherlands, Norway and Switzerland, there was even a trend that TTR was longer in medicines with an AA status. A challenge with AA status is that the level of evidence for clinical benefit is often low, leading to reimbursement difficulties. In our study, four out of five of AA-graded anticancer medicines had a low ESMO-MCBS level.

For the country-related factor, Germany seemed an outlier being the largest country with the highest GDP and no pre-assessment procedure, resulting in a TTR in all anticancer medicines shorter than 5 days after EU-MA (Figure 1). The system of early market access in Germany is based on the Act on the Reform of the Market for Medical Products (AMNOG).^13^ This is an approach to assess additional benefits of new drugs launched in the German market and pricing according to their efficacy. After 1 year of its use, if no additional benefit is found, the pharmaceutical is allocated to a reference price group with comparable activity. A major drawback of this system is that prices are very high. This is the reason why the system will be adjusted. Evaluation will take place sooner (after 6 months) and will be judged more rigorously.^22^ In all other countries where P&R does play a role, TTR is longer. In our study we also observed a linear association between GDP and TTR. From the literature it is known that a high GDP or a high expenditure on health per capita is associated with a short(er) TTR.^7^, ^8^, ^9^, ^10^ These findings can be explained by the fact that countries with a higher GDP have higher numbers of citizens and thus higher numbers of patients. Higher numbers of patients mean a potential larger market. Secondly, countries with a higher GDP have probably a higher HTA capacity. At last, countries with a higher GDP can afford higher health care expenditure and price negotiations might be perceived as less important in relation to accessibility. Each HTA makes its own assessment and has its own assessment criteria (e.g. QALY in UK,^23^ PASKWIL in the Netherlands^24^), and the assessment may be based on cost-effectiveness (e.g. UK) or magnitude of clinical benefit (e.g. Germany) leading to different interpretations. This multinational process is time- and resource-consuming and the harmonization process within the EU is progressing slowly, although initiatives for collaboration and joint HTA reports have been initiated including EUnetHTA and Beneluxa.^25^

The indication-related factors assessed in our study, orphan medicines and CMA-approved anticancer medications, both seemed to be reimbursed slower than that of non-orphans and non-CMA medications. Given the high price and uncertain long-term benefit of many orphan drugs, we suggest that prolonged P&R negotiations may contribute to potential slower reimbursement decisions. The reason for slower reimbursement for CMA medicines might be that it is difficult to determine the added value of the medicine by HTA and the criteria are not yet well established. Without the support of HTA bodies and payers, the uptake of adaptive pathways such as CMA remain limited.^26^ Moreover, ending reimbursement is more difficult than not starting reimbursement in the first place, both for policymakers and for the public.^27^ Longer TTR for CMA-approved medicines in some countries is a non-significant but important finding in our study, and criteria must be drawn up for reimbursement, preferentially at an international level.

In our study we found a shorter TTR for medicines submitted by a Big-12 pharmaceutical company. We did not examine the reason for this and it is not described in the literature. An explanation for this might be that large pharmaceuticals can commit more resources and have a better infrastructure for research, preparation before HTA and marketing. Moreover, Big-12 pharmaceutical companies obviously have a larger portfolio of medicines and their existence is not depending on just one or a few products.

### Limitations of the study

This study has a number of limitations. Firstly, this study was based on data from several publicly available internet websites. The indicators we adopted to measure availability and access to new anticancer medicines have some limitations. The publication of HTA reimbursement decision does not guarantee the actual availability of medicines to patients. Because after the decision of reimbursement, several steps need to be taken before the new anticancer medicine can be prescribed by oncologists, i.e. inclusion in the health insurance package, inclusion in medical guidelines, reimbursement agreements between health insurers and hospitals, purchase of the medicines by the hospitals, admission to the hospital formulary and informing prescribers. As we are interested only in publicly available medicines, we did not examine mechanisms other than HTA approvals for patients to get access to medicines like individually arranged access as in open extensions of pivotal clinical trials or compassionate-use or early-access programs (EAPs).

Secondly, because we limited this study to new anticancer medicines for solid tumors, the number of medicines examined is restricted and hinders statistical analysis. We were interested in TTR in high-income countries, but this selection limits generalizability. We did not examine the post-reimbursement access time and the EAP as we considered these programs are not accessible to all cancer patients. Furthermore, we did not quantify prices.

Finally, several other country-based factors could play a role that we could not examine in the context of this study, for example, coverage decisions that consider cost-effectiveness,^28^, ^29^, ^30^, ^31^, ^32^ acceptability of intermediate outcomes, P&R negotiations,^7^^,^^33^^,^^34^ interdependencies in pricing among countries, the budget impact of introduction,^7^^,^^35^ the capacity and complexity of the national reimbursement system^30^ and the market size.^7^

Faster reimbursement of anticancer medicines is one of the markers of regulatory success, but approval of drugs that improve OS has steadily declined over the years.^36^^,^^37^ The reason for this may be that PFS and disease-free survival (DFS) are accepted endpoints for EMA. An advantage of PFS and DFS as endpoint might be earlier available trial results; moreover, OS cannot always be documented due to sequential therapies. However, disadvantages of these endpoints include uncertain evidence about their clinical value and the uncertain correlation between the surrogate endpoints and, for instance, OS as the main outcome.^37^ The level of evidence might be an equally important aspect for regulatory focus compared to approval of the number of drugs and the TTR. Since approval status and TTR does not always translate into a clinical benefit, stakeholders and decision-makers should focus on the benefit/risk ratio of anticancer drugs to ensure an appropriate allocation of resources in health care systems.^38^

Further recommendations to improve the access of patients to novel anticancer medicines have been formulated by a multidisciplinary group from pharmaceutical companies, academia and (post) regulatory agencies in 2007, including earlier assistance from regulators and to make better use of registries and post-marketing studies.^39^ Examples of such changes in recent years in the development, licensure and appraisal process are early scientific advice and regulatory guidance for pharmaceutical companies by EMA and national regulatory authorities and the introduction of conditional reimbursement (with the obligation to gather more data). Furthermore, initiatives for cross-border collaboration and joint HTA reports have originated such as Beneluxa and EUnetHTA.^25^^,^^40^

This study shows that even high-income countries are facing the challenge to safeguard quick and affordable access and reimbursement of new/innovative anticancer medicines. Given the apparent importance of GDP as a factor contributing to a short TTR, it would be a suggestion to pool resources for smaller countries. Ultimately, a joint European assessment for reimbursement could be an efficient approach, so that not all national HTA bodies with different processes, criteria and limited capacity have to do the same time-consuming job. Criteria have to be drawn up for the accepted level of evidence and reimbursement and need to be updated regularly. One particular issue for debate would be what we as a community would want to pay for access to innovative medicines. The development of a united price model by all stakeholders, for a fair price that promotes accessibility, depending on the added value of a drug that is affordable and stimulates innovation, is essential. If proof of efficacy is difficult to obtain, for example, because of rarity of the cancer or uncommon mutations, data should be collected internationally. To save time, processes of rating and P&R should be parallelized with the EU-MA process.

### Conclusion

Future demographic changes in the world’s population will cause cancer to become a growing public health problem in Europe and worldwide. To face this challenge, improving anticancer drug development and the efficiency of health systems is a top priority. More efficacious anticancer medicines must reach patients in a timely fashion and at affordable prices.

However, TTR of innovative anticancer medicines is variable and too long in most countries. This leads to inequality in access even between high-income countries. The majority of factors examined in this study (ESMO-MCBS, and orphan, CMA and AA status) all seem to be of minor importance. The only significant factors seemed the country-specific GDP, the absence of a pre-assessment procedure (which has its own disadvantages) and submission by big pharmaceutical companies.

Altogether, these findings suggest delay in TTR among high-income countries is most likely caused by the process of HTA, P&R negotiations and the NRA systems. Solutions to shorten TTR most likely include measures to harmonize HTA assessments, P&R negotiations and the NRA systems, especially between (smaller) countries with a lower GDP.

## Supplementary data

Appendix 1 Data collection and selection of anticancer medicines

Appendix 2 Details of Statistical analysis

Appendix 3 Detailed overview of time to reimbursement for the different factors in the different countries

Heatmap Appendix Heatmap figure of time to reimbursement for the different medicines in the different countries
