# Supplementary material for: Time to reimbursement of novel anticancer drugs in Europe: a case study of seven European countries
Source: ESMO Open. 2023 Apr 6;8(2):101208. doi: 10.1016/j.esmoop.2023.101208 (PMC10163159; doi:10.1016/j.esmoop.2023.101208)
Supplement: Appendix 1 — Data collection and selection of anticancer medicines [file mmc1.docx]

**Appendix 1 – Data collection and selection of anticancer medicines**

| **The relevant Health Technology Assessment (HTA) - and reimbursement websites for each country and methods for data extraction** | |
| --- | --- |
| Germany: | PharmNet.Bund - Arzneimittel-Informationssystem (pharmnet-bund.de)   - Go to “drug information system” - Click on “search access” - Click on “Akzeptieren und weiter zur Recherche” - Enter the generic name in the search box “suche nach” - Click on “stoffname” in the drop down menu and press enter. - Click on the - The date of access is entitled “Datum der Zulassung/Registrierung (Wirksamkeitsdatum)” |
| The United Kingdom | [Published guidance, NICE advice and quality standards \| Guidance \| NICE](https://www.nice.org.uk/guidance/published?)   - Enter the generic name in filter by title in keyword search box - Date of access extracted is date of the acknowledged Technology Appraisal Guidance |
| France | <http://agence-prd.ansm.sante.fr/php/ecodex>;   - Enter the trade name in the field drop down menu - Click on “ok” - “spécialités pharmaceutiques répondent à vos critères” appears - Click on the “trade names” - The date of access is entitled “date de láutorisation” - Check for the oldest date of access and - Check for “Statut de l'autorisation : **VALIDE”** |
| Belgium | [https://webappsa.riziv-inami.fgov.be/SSPWebApplicationPublic/nl/Public/ProductSearch](https://eur04.safelinks.protection.outlook.com/?url=https%3A%2F%2Fwebappsa.riziv-inami.fgov.be%2FSSPWebApplicationPublic%2Fnl%2FPublic%2FProductSearch&data=05%7C01%7Ch.c.post%40amsterdamumc.nl%7Cab49819e6c364c82288408daef3c18dc%7C68dfab1a11bb4cc6beb528d756984fb6%7C0%7C0%7C638085338756776111%7CUnknown%7CTWFpbGZsb3d8eyJWIjoiMC4wLjAwMDAiLCJQIjoiV2luMzIiLCJBTiI6Ik1haWwiLCJXVCI6Mn0%3D%7C3000%7C%7C%7C&sdata=pW5XYYYssWEr0DIMYmHGSRfnO1trWR%2BV6%2BnleKTK3ik%3D&reserved=0)   - Go to “eenvoudig zoeken” - Enter the trade name in the search box “product” - Click on “zoeken” - The product appears - Click on the “trade names” - Search for “details van het product”   The date of access is entitled “het product wordt terugbetaald sinds” |
| Norway | <https://nyemetoder.no>   - Enter the trade name in the search box “sØk i alt innhold” - Click on “search” - The product appears - Read the outcomes with a translation program (google-translate) - Search for “beslutning” - Information on the product and dates are found in the text |
| Switzerland | <https://www.swissmedic.ch>   - Enter the trade name in the search box “suchbegriffe” - Click on “search” - The product appears - Read the outcomes - Search for “zulassungen” - The date of access is entitled “zulassungsdatum: ….”   and <http://www.spezialitaetenliste.ch>   - Go to “spezialitaetenliste” - Click on “[Präparatsuche nach Name](https://www.spezialitaetenliste.ch/ShowPreparations.aspx" \o "Präparatsuche nach Name (mit Geburtsgebrechen-Spezialitätenliste)) ” - The “Präparate Spezialitätenliste” appears - Enter the trade name in the search box “suchtext” - The date of access is entitled “Aufnahme” |
| The Netherlands | As there is no specific website for the Netherlands, therefore we used the historical G-standard. This G-standard is not publicly available and is monthly send to the financial departments of the Dutch hospitals. It contains all the EU-MA medicines with their first reimbursement dates per indication. |

Access date: 1/1/2023

**Flow diagram of the identification and selection of anticancer medicines**

**Identification of anticancer medicines**

Records removed *before screening*:

Generic medicines, hybride medicines⃰, informed consent applications⃰⃰ ⃰ and biosimilars

(n = 161)

Positive recommendations on new medicines (n=419)

**Identification**

New medicines for first indication (n = 258)

Non hemato-oncological medicines excluded

(n = 198)

Anticancer medicines for solid and haemological tumours

(n =65 )

Malignant haematological medicines excluded

(n =23)

**Screening**

Anticancer medicines for solid tumours (n =42 )

Medication excluded:

Diagnostic (n = 3)

Symptom treatment (n = 2)

Existing medication (n = 1)

Withdrawn by EMA (n=1)

Anticancers medicines included

(n = 35)

**Included**

.⃰ Hybrid applications rely in part on the results of pre-clinical tests and [clinical trials](https://www.ema.europa.eu/en/glossary/clinical-trial) for a reference product and in part on new data.

⃰ ⃰ An [informed consent application](https://www.ema.europa.eu/en/glossary/informed-consent-application) makes use of data from the dossier of a previously authorised medicine, with the [marketing authorisation holder](https://www.ema.europa.eu/en/glossary/marketing-authorisation-holder) of that medicine giving consent for the use of their data in the application.

⃰ ⃰⃰ ⃰ A [biosimilar medicine](https://www.ema.europa.eu/en/glossary/biosimilar-medicine) is a [biological medicine](https://www.ema.europa.eu/en/glossary/biological-medicine) that is highly similar to another [biological medicine](https://www.ema.europa.eu/en/glossary/biological-medicine) that is already authorised for use

*The flow diagram was adapted from* Page MJ, McKenzie JE, Bossuyt PM, Boutron I, Hoffmann TC, Mulrow CD, et al. The PRISMA 2020 statement: an updated guideline for reporting systematic reviews. BMJ 2021;372:n71. doi: 10.1136/bmj.n71
