# Supplementary material for: Time to reimbursement of novel anticancer drugs in Europe: a case study of seven European countries
Source: ESMO Open. 2023 Apr 6;8(2):101208. doi: 10.1016/j.esmoop.2023.101208 (PMC10163159; doi:10.1016/j.esmoop.2023.101208)
Supplement: Appendix 2 — Details of Statistical analysis [file mmc2.docx]

**Appendix 2**

**Difference in TTR between the countries.**

There is a statistically significant difference in TTR between the different countries (p<0.001) When the differences between countries are considered in pairs, statistically significant differences remain between countries with shorter and longer TTR for correction. Even after adjusting for multiple testing according to Bonferroni (see section Bonferroni correction) statistically significant differences remain.

|  | GE | FR | UK | NL | NO | CH | BE |
| --- | --- | --- | --- | --- | --- | --- | --- |
| GE |  |  |  |  |  |  |  |
| FR | <.001* |  |  |  |  |  |  |
| UK | <.001* | .376 |  |  |  |  |  |
| NL | <.001* | .455 | .890 |  |  |  |  |
| NO | <.001* | <.001* | .006 | .004 |  |  |  |
| CH | <.001* | <.001* | .009 | .006 | .912 |  |  |
| BE | <.001* | <.001* | <.001* | <.001* | .472 | .407 |  |

p ≤.05  in green. * p ≤.05 after Bonferroni correction (adjusted p<0.0024 see below, Bonferroni correction)

| **Paired Mann-Whitney U Test of TTR within countries between presence or absence of a factor.** | | | | | | |
| --- | --- | --- | --- | --- | --- | --- |
|  | ESMO MCSB | Orphan medicine status | Big 12 | Accelarated approval status | Conditional marketing authorisation status |  |
| GE | .010 | .446 | .781 | .873 | .067 |  |
| FR | .821 | .516 | .125 | .945 | .107 |  |
| UK | .880 | .056 | .612 | .945 | .636 |  |
| NL | .970 | .492 | .301 | .369 | .390 |  |
| NO | .182 | .005* | .056 | .802 | .299 |  |
| CH | .404 | .086 | <.001* | .237 | .612 |  |
| BE | .223 | .101 | .004* | .477 | .299 |  |

p ≤.05  in green. * p ≤.05 after Bonferroni correction (adjusted p<0.0071 see below, Bonferroni correction)

**Linear regression (GDP vs TTR), excluding Germany**

Best-fit values

Slope -0.0002119 (SD 4.094e-005; 95% CI -0.0003171 to -0.0001066)

Y-intercept 727.2 (SD 78.66; 95% CI 525.0 to 929.4)

X-intercept 3432347 (95% CI 2638573 to 5469402)

1/slope -4720

Goodness of Fit

R squared 0.8427; P 0.0035; Equation Y = -0.0002119*X + 727.2

**Bonferroni correction**

Given the number of statistical tests performed within this study we used a Bonferroni correction. For this correction we corrected for individual comparisons between countries and within the different countries between presence/absence of the factors.

The correction used for the paired differences between the countries was the number of individual paired comparisons (n=21). Therefore the level to reach significance ( p<0.05) was adjusted to 0.05/21 = p<0.0024

The correction used for the Paired Mann-Whitney U Test of TTR within countries between presence or absence of a factor was the number of countries (7) minus 1. Therefore the level to reach significance ( p<0.05) was adjusted to 0.05/(7-1) = p<0.0071.
