# Supplementary material for: Time to reimbursement of novel anticancer drugs in Europe: a case study of seven European countries
Source: ESMO Open. 2023 Apr 6;8(2):101208. doi: 10.1016/j.esmoop.2023.101208 (PMC10163159; doi:10.1016/j.esmoop.2023.101208)
Supplement: Appendix 3 — Detailed overview of time to reimbursement for the different factors in the different countries [file mmc3.docx]

**Time to reimbursement of novel anticancer drugs in Europe, a case study of seven European countries**

**Appendix 3 – Detailed overview of time to reimbursement for the different factors in the different countries**

|  |  | Germany | | | France | | | The United Kingdom | | | The Netherlands | | | | Norway | | | | Switzerland | | | Belgium | | |
| --- | --- | --- | --- | --- | --- | --- | --- | --- | --- | --- | --- | --- | --- | --- | --- | --- | --- | --- | --- | --- | --- | --- | --- | --- |
|  |  | Median | Range | IQR | Median | Range | IQR | Median | Range | IQR | Median | Range | IQR | Median | | Range | IQR | Median | | Range | IQR | Median | Range | IQR |
| ESMO MCSB | Low (n=19) | 3 | 1 - 4 | 2 - 3 | 189 | 37 - 1956 | 68 - 552 | 251 | 12 - 2270 | 134 - 539 | 310 | 71 - 1010 | 118 - 456 | 656 | | 274 - 2270 | 483 - 958 | 713 | | 74 - 2270 | 524 - 1105 | 829 | 317 - 1956 | 553 - 1014 |
|  | High (n=13) | 4 | 1 - 5 | 3 - 4 | 107 | 32 - 2320 | 91 - 341 | 238 | 40 - 1291 | 160 - 446 | 340 | 72 - 1584 | 252 - 494 | 536 | | 135 - 2320 | 401 - 628 | 438 | | -81 - 2320 | 278 - 1222 | 643 | 162 - 2320 | 350 - 752 |
| Accelerated approval status | non-Accelerated approval (n=30) | 3 | 1 - 5 | 2 - 4 | 166 | 0 - 1956 | 84 - 552 | 254 | 12 - 2270 | 160 - 539 | 343 | 71 - 1878 | 118 - 553 | 627 | | 135 - 2270 | 483 - 829 | 653 | | -81 - 2270 | 347 - 1222 | 747 | 162 - 1956 | 408 - 1059 |
|  | Accelerated approval (n=5) | 3 | 2 - 4 | 3 - 3 | 107 | 62 - 2320 | 72 - 524 | 334 | 128 - 517 | 251 - 337 | 494 | 173 - 743 | 309 - 713 | 666 | | 335 - 2320 | 409 - 1200 | 986 | | 599 - 2320 | 713 - 1344 | 713 | 479 - 2320 | 560 - 1923 |
| Orphan Medicine status | non-Orphan medicine (n=26) | 3 | 1 - 5 | 3 - 4 | 130 | 0 - 2320 | 68 - 524 | 241 | 12 - 1878 | 134 - 446 | 343 | 71 - 1878 | 158 - 494 | 560 | | 135 - 2320 | 409 - 666 | 644 | | -81 - 2320 | 347 - 1105 | 689 | 162 - 2320 | 408 - 961 |
|  | Orphan medicine (n=9) | 3 | 1 - 4 | 2 - 3 | 189 | 62 - 1503 | 107 - 552 | 471 | 205 - 2270 | 251 - 829 | 372 | 74 - 1503 | 227 - 723 | 1200 | | 335 - 2270 | 829 - 1503 | 1135 | | 74 - 2270 | 829 - 1503 | 1059 | 317 - 1923 | 829 - 1503 |
| Conditional marketing authorisation status | no conditional marketing authorisation (n=24) | 3 | 2 - 5 | 3 - 4 | 347 | 0 - 2320 | 92 - 573 | 289 | 118 - 2270 | 161 - 631 | 310 | 71 - 1878 | 166 - 475 | 593 | | 135 - 2320 | 398 - 864 | 653 | | -81 - 2320 | 392 - 1544 | 909 | 162 - 2320 | 444 - 1459 |
|  | Conditional marketing authorisation (n=11) | 2 | 1 - 4 | 1 - 4 | 89 | 32 - 884 | 62 - 189 | 265 | 12 - 829 | 128 - 538 | 444 | 74 - 743 | 118 - 619 | 666 | | 494 - 1684 | 536 - 1200 | 713 | | 74 - 1222 | 383 - 986 | 689 | 317 - 1684 | 560 - 829 |
| Company size Big12 | No (n=19) | 3 | 1 - 4 | 2 - 4 | 341 | 0 - 2320 | 103 - 622 | 337 | 40 - 2270 | 160 - 539 | 373 | 71 - 1878 | 173 - 713 | 666 | | 335 - 2320 | 536 - 1584 | 1135 | | 437 - 2320 | 623 - 1878 | 1014 | 407 - 2320 | 643 - 1684 |
|  | Yes (n=16) | 3 | 1 - 5 | 3 - 4 | 92 | 37 - 1370 | 65 - 405 | 247 | 12 - 1291 | 180 - 472 | 297 | 74 - 1010 | 138 - 436 | 577 | | 135 - 1215 | 398 - 697 | 365 | | -81 - 1370 | 139 - 707 | 583 | 162 - 1370 | 334 - 818 |
| Pre-assessment procedure | Present | - | - | - | 142 | 0 - 2320 | 72 - 552 | 265 | 12 - 2270 | 160 - 538 | 345 | 71 - 1878 | 158 - 567 | 628 | | 135 - 2320 | 420 - 958 | 689 | | -81 - 2320 | 383 - 1344 | 742 | 162 - 2320 | 479 - 1370 |
|  | Absent | 3 | 1 - 5 | 2 - 4 | - | - | - | - | - | - | - | - | - | - | | - | - | - | | - | - | - | - | - |

**Appendix 3:** Complete dataset of Time to reimbursement for the different factors in the different countries, accept for pre-assessment procedure. The data is the Censored data where the censoring date of 01/01/2023 was used. Data was complete for all included medicines (n=35) for all factors accept ESMO MCSB score. Three drugs were not assessed by ESMO and no ESMO-MCBS score was available.
