# Supplementary figures and images for: Time to reimbursement of novel anticancer drugs in Europe: a case study of seven European countries
Source: ESMO Open. 2023 Apr 6;8(2):101208. doi: 10.1016/j.esmoop.2023.101208 (PMC10163159; doi:10.1016/j.esmoop.2023.101208)

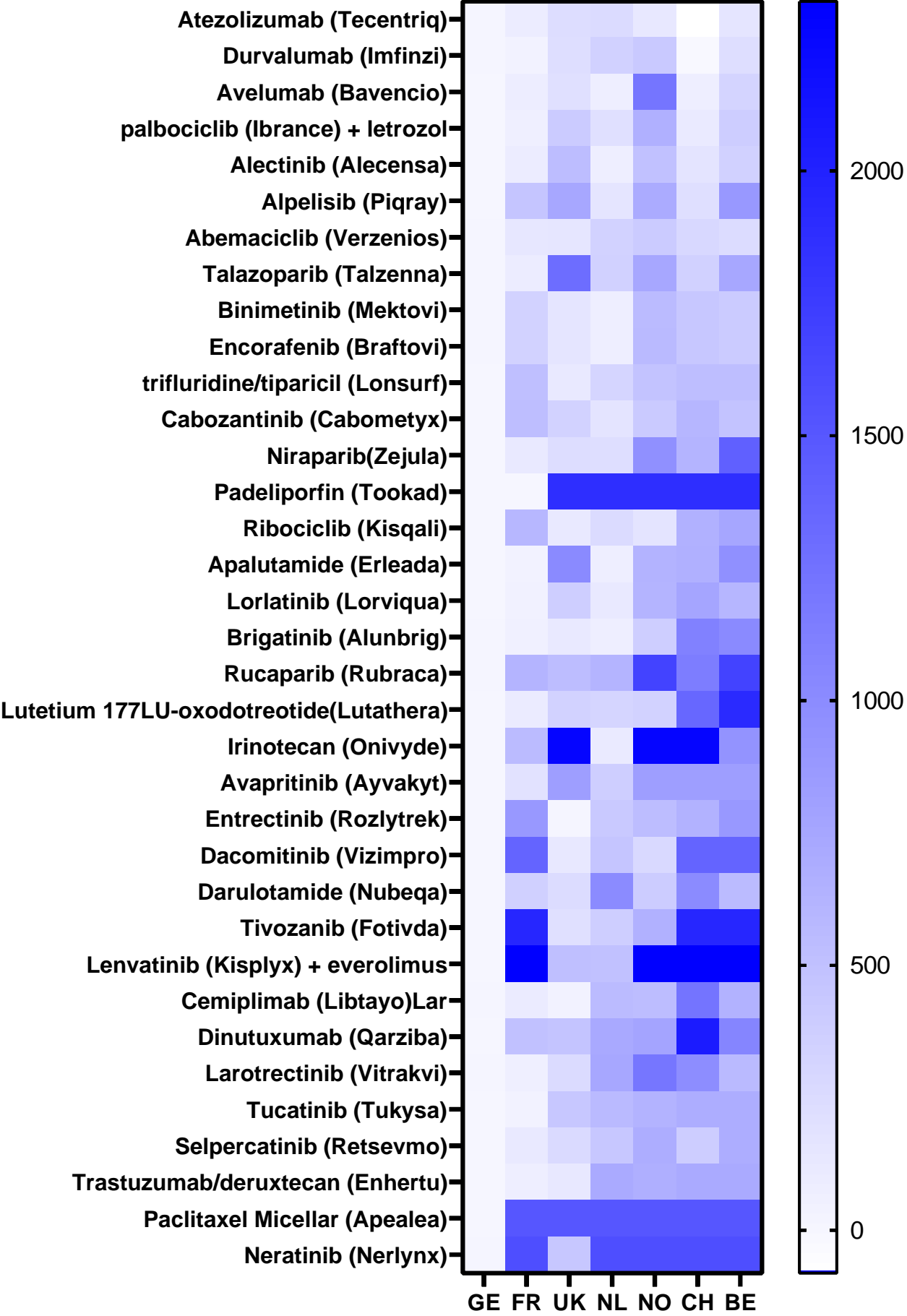

Supplement: Heatmap Appendix — Heatmap figure of time to reimbursement for the different medicines in the different countries [file mmc4.pdf]
